# A case of intramandibular neurofibroma resembling a radicular cyst in a neurofibromatosis type 1 patient

**DOI:** 10.1016/j.ijscr.2021.105883

**Published:** 2021-04-10

**Authors:** Yuki Kunisada, Norie Yoshioka, Soichiro Ibaragi, Tatsuo Okui, Hitoshi Nagatsuka, Akira Sasaki

**Affiliations:** a Department of Oral and Maxillofacial Surgery, Okayama University Graduate School of Medicine, Dentistry and Pharmaceutical Sciences, Okayama, Japan; b Department of Oral and Maxillofacial Surgery, Faculty of Medicine, Shimane University, Izumo, Shimane, Japan; c Department of Oral Pathology and Medicine, Okayama University Graduate School of Medicine, Dentistry and Pharmaceutical Sciences, Okayama, Japan

**Keywords:** Mandible, Multiple café-au-lait spots, Neurofibromatosis type 1

## Abstract

**Introduction:**

Neurofibromatosis is a disease that causes various abnormalities such as neurofibroma, mainly in the skin and nerves. The common sites in the oral cavity are the palate, gingiva, tongue, buccal mucosa, and lips but, occurrence in the mandible is rare.

**Presentation of case:**

A 26-year-old woman was referred to our clinic because of percussion pain. Radiographic findings showed a radiolucent area. The patient was clinically diagnosed with a radicular cyst by a previous doctor. Multiple café-au-lait spots were found disseminated on her body, and she had already been prenatally diagnosed with neurofibromatosis type 1 (NF1). We performed a biopsy and suggested a neurofibroma. Tumor extirpation was performed under general anesthesia. The histopathological diagnosis showed a neurofibroma.

**Clinical discussion and conclusion:**

NF1 is a systemic nevus that causes abnormalities in melanocytes and Schwann cells, and various lesions appear, but intramandibular lesions are extremely rare. Diagnosis of NF1 and radicular cysts in the mandible is difficult due to their image resemblance. However, it should be kept in mind if the underlying disease is NF1. In our case, it was easy to detach and may have originated from small peripheral nerve endings in the mandible.

## Highlights

• Neurofibromatosis is a disease that causes various abnormalities such as neurofibroma, mainly in the skin and nerves.
• The common sites of occurrence in the oral cavity are the palate, gingiva, etc., occurrence in the mandible is rare.
• The patient had already been prenatally diagnosed with neurofibromatosis type 1.
• The lesion was clinically diagnosed with a radicular cyst, but histopathological diagnosis showed a neurofibroma.
• Tumor extirpation was performed under general anesthesia.

## 1 Introduction

Neurofibromatosis type 1 (NF1) is also known as von Recklinghausen's disease. It is an autosomal dominant inherited disorder whose main symptom is neurofibroma. It frequently occurs in the characteristic skin pigment spots, skin, and nerves [1]. Neurofibroma is less frequently expressed in the oral cavity as a symptom of this disease, especially in cases occurring in the mandible. We report a case of neurofibroma of the mandible resembling a radicular cyst.

## 2 Methods

Written informed consent was obtained from the patient for publication of this case report and accompanying images. A copy of the written consent is available for review by the Editor-in-Chief of this journal on request. This research work has been reported in line with the SCARE 2020 criteria [2].

## 3 Presentation of case

**Fig. 1:**
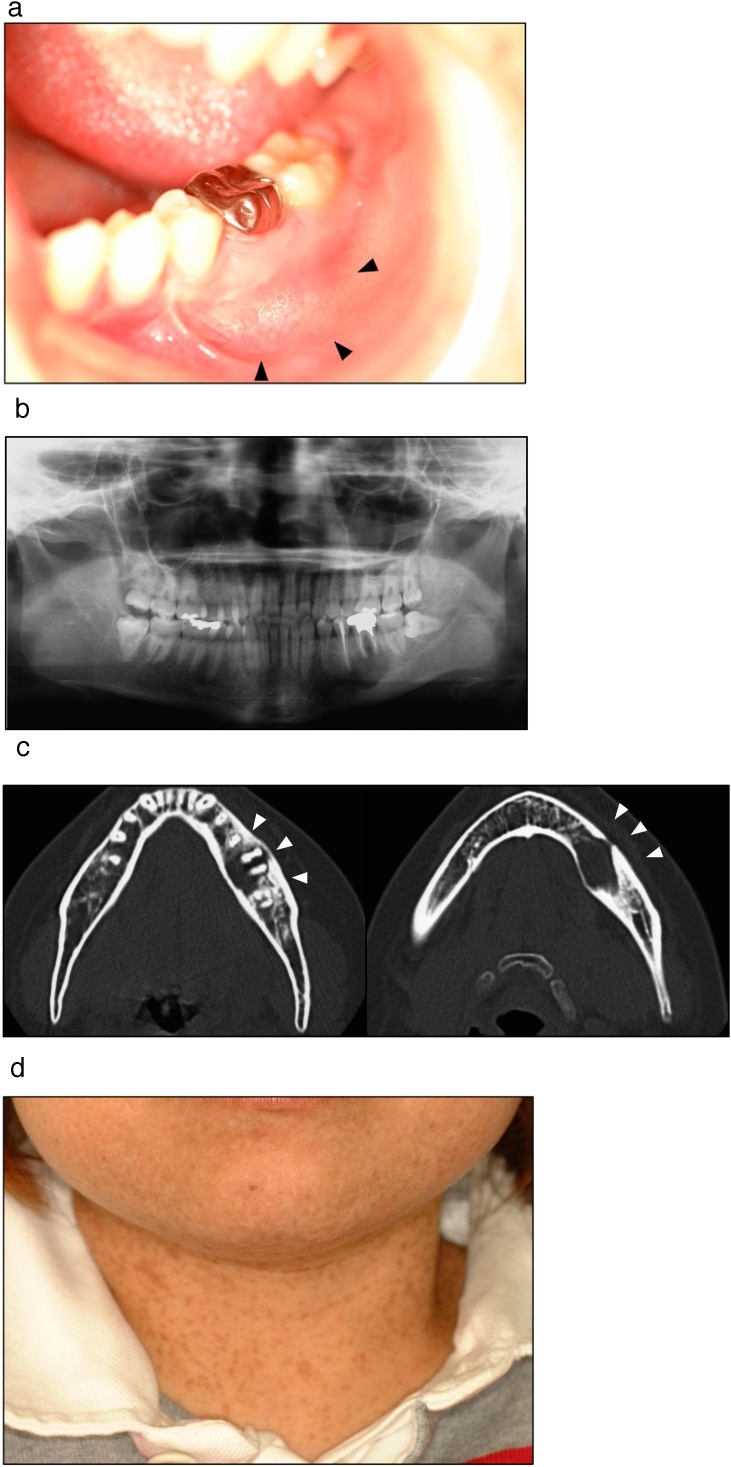
a) Intraoral findings; A bulge of the bone-like hardness of approximately 35 × 20 mm was observed at the gingivobuccal fold of the left mandibular molar. b) Panoramic radiograph showing a well-defined mass in the left mandibular molar region. c) A 14 × 21-mm region was observed in the left mandible molar on CT images. d) Numerous cafe au lait spots on the facial of the skin.

A 26-year-old female patient was referred to our department by her primary care dentist because of percussion pain in the left lower first molar. Her face was symmetrical, and she had no paraesthesia in her left mental region. She had a bone-like hard swelling in the gingivobuccal fold from the left lower second premolar to the first molar (Fig. 1a). Her left lower first molar had degree 1 mobility. Radiographic findings showed a radiolucent area around the root apex of the left lower first molar, and the inferior alveolar nerve was excluded by the lesion (Fig. 1b). On computed tomography (CT), the lesion was continuous with the periodontal cavity of the left lower second premolar and the first molar, the boundary between the lesion and bone was clear, and the inside of the lesion showed uniform radiolucency (Fig. 1c,d). The patient was clinically diagnosed with a radicular cyst by a previous doctor.

The patient had multiple café-au-lait spots disseminated on her body. These spots were also observed on the facial skin (Fig. 1e). Her chest X-ray image showed scoliosis, but no abnormalities in the central nervous system were observed. She had been diagnosed with NF1 prenatally and was undergoing observation follow-up once a year after the orthopedic surgery. There was no drug history and allergies, and no family history of NF1 infection.

A biopsy was performed under local anesthesia during the first visit. Histological examination of biopsies revealed that most of them were inflammatory granulation tissue. In addition, spindle-shaped cells with wavy nuclei along with collagen fibers, some of which were positive for S-100 protein, suggesting a possibility of neurofibroma. We strongly suspected it to be a neurofibroma from her medical history.

Under general anesthesia, we performed the extraction of the left lower first molar and tumor extirpation and apicoectomy of the left lower second premolar. There was no adhesion between the tumor and the surrounding bone. A part of the bone wall of the mandibular canal was missing, and a lower alveolar neurovascular bundle was observed. The patient’s prognosis was favorable with no signs of recurrence after the operation.

**Fig. 2:**
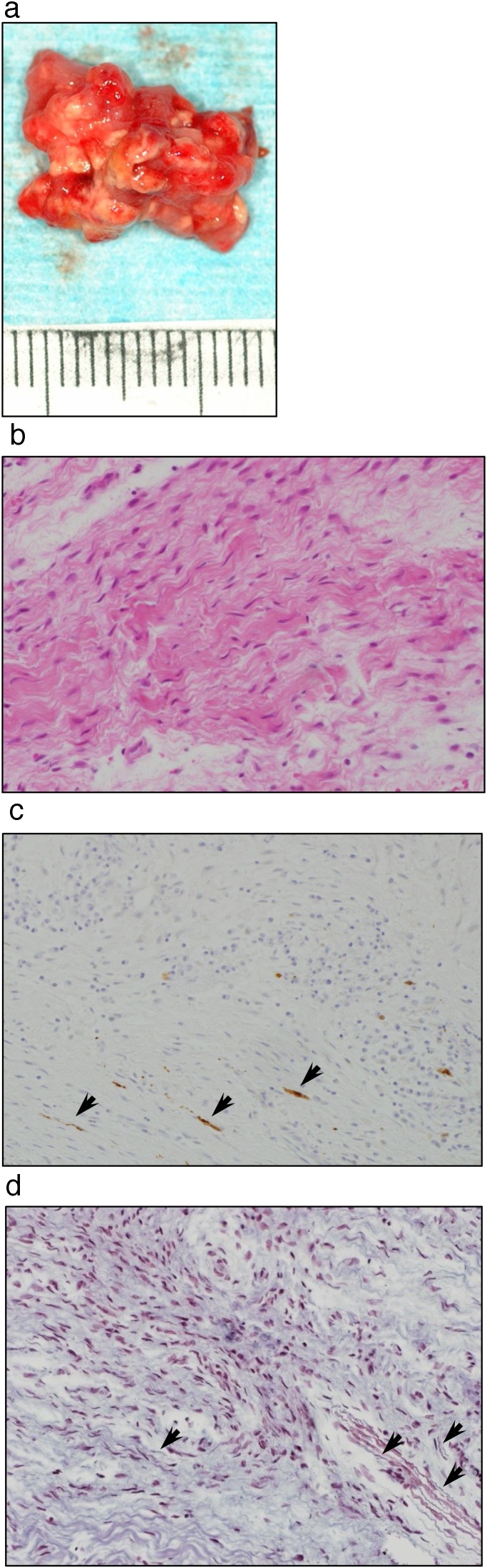
a) The excised specimen was 16 × 13 × 13 mm, elastically soft, and had heterogeneous granulation-like tissue with a mixture of milky white and red tissues. b) H&E staining (× 400) showed marked proliferation of spindle cells with corrugated nuclei along with fibroblasts and collagen fibers. c) Immunohistochemical staining (× 400) for S-100 protein (→) shows positive tumor cells scattered in the fibrous connective tissue. d) Bodian's staining (× 400) showed dark brown fibers (→) in connective tissue that were suspected to be neurofibrils.

The extracted specimen was 16 × 13 mm elastic-soft mass with heterogeneous granulation-like tissue with a mixture of milky white and red tissues (Fig. 2a). Histopathological findings showed marked proliferation of spindle cells with corrugated nuclei along with fibroblasts and collagen fibers by H&E staining (Fig. 2b). Immunohistochemically, S-100 protein-positive tumor cells were found scattered in the fibrous connective tissue. The Bodian's staining showed dark brown fibers in the connective tissue that were suspected to be neurofibrils (Fig. 2c,d). Cytokeratins AE1/AE3 were negative. A comprehensive diagnosis of neurofibromas associated with NF1 was made, including pathological findings.

## 4 Clinical discussion and conclusion

Neurofibromatosis is a disease that causes various abnormalities such as neurofibroma, mainly in the skin and nerves. It is classified into neurofibromatosis type 1 and neurofibromatosis type 2 (NF2). NF1 has systemic café-au-lait spots and neurofibromas, and NF2 has bilateral acoustic neuroma. The causative genes of NF1 and NF2 are located on chromosome 17 (17q11.2) and chromosome 22, respectively, and the causative genes are completely different [3,4]. NF1 was first reported by Friedrich Daniel von Recklinghausen in 1882 and is also known as von Recklinghausen's disease. NF1 is a systemic nevus that causes abnormalities in melanocytes and Schwann cells, and various lesions appear on the skin, nervous system, eyes, bones, etc., with age, in addition to café-au-lait spots and neurofibromas [5]. The current NF1 diagnostic criteria in 1987 at the National Institute of Health Consensus Development Conference is two or more of the following manifestations: (1) six or more café au lait spots (≥ 5 mm in children, ≥ 15 mm in adults), (2) two or more neurofibromas of different types or plexiform neurofibromas; (3) axillary or inguinal lentigines; (4) glioma of the nervus opticus; (5) one or more Lisch nodules; (6) bone disorders; and (7) familiarity for NF1 diagnosed as above [6]. Our case corresponds to criteria (1) and (6), and NF1 was already diagnosed in the pediatrics department of another hospital. Therefore, genetic testing was not performed in our hospital. NF1 is autosomal dominant, but the NF1 gene is frequently mutated, and 50% of patients have sporadic mutations in Japan [7]. Approximately 7% of neurofibromas have been reported in the oral cavity as a symptom of NF1 [8,9]. The common sites in the oral cavity are the palate, gingiva, tongue, buccal mucosa, and lips [10]. Occurrence in the mandible is rare, and most cases are accompanied by bone deformation [11]. In particular, NF1 cases with histopathologically diagnosed neurofibromas with intramandibular lesions are extremely rare [12]. Vivian et al. reported that approximately 15% of female patients with NF1 had periapical cemental dysplasia (PCD) [13]. In our case, a lesion in the mandible was clinically diagnosed as a radicular cyst by a previous doctor. For this reason, she received root canal treatment, and inflammatory modification was observed in the lesion, and it was difficult to obtain a typical histopathological finding of neurofibroma. PCD does not require treatment, but neurofibromas require extirpation. Imaging examination by CT or magnetic resonance may contribute to distinguishing between the two lesions [14].

Neurofibromas occurring in the mandible may be derived from the inferior alveolar nerve main duct or small peripheral nerve endings. Because the tumor does not have a capsule and is prone to recurrence, resection of the inferior alveolar nerve vascular bundle is indicated when the tumor is derived from the inferior alveolar nerve [15]. However, when it originates from the peripheral nerve, the inferior alveolar neurovascular bundle is excluded by the tumor, and it is usually easy to detach from the tumor. In our case, the bone wall of the mandibular canal was partially lost. However, the inferior alveolar nerve did not adhere to the tumor and was easily detached, and widening of the mandibular canal and other bone defects were not observed. This suggests that it may have originated from peripheral nerves. After the operation, due to the patient's convenience, she was followed up by imaging examination every few months at our affiliated hospital. She visited our hospital for other treatments, and more than 10 years have passed, but no evidence of recurrence has been observed.

## Declaration of Competing Interest

None.

## Sources of funding

None.

## Ethical approval

The study was approved by the Okayama University Graduate School of Medicine, Dentistry and Pharmaceutical Sciences and Okayama University Hospital, Ethics Committee.

## Consent

Formal consent was obtained from the patient for the publication of this case report and any accompanying images. A copy of the written consent is available for review by the Editor-in-Chief of this journal on request.

## Author’s contribution

Yuki Kunisada: Main author, contributing in study concept/design, data collection, analysis, and writing the paper.

Norie Yoshioka: Investigation, resources, writing - review & editing.

Soichiro Ibaragi: Validation, supervision.

Tatsuo Okui: Validation, supervision.

Hitoshi Nagatsuka: Histopathology, data interpretation, and editing the paper.

Akira Sasaki: Validation, supervision.

## Registration of research studies

Not applicable.

## Guarantor

Akira Sasaki professor.

## Provenance and peer review

Not commissioned, externally peer-reviewed.
